# Multiple gene genealogy reveals high genetic diversity and evidence for multiple origins of Chinese *Plasmopara viticola* population

**DOI:** 10.1038/s41598-017-17569-7

**Published:** 2017-12-11

**Authors:** Wei Zhang, Ishara S. Manawasinghe, Wensheng Zhao, Jianping Xu, Siraprapa Brooks, Xueyan Zhao, Kevin D. Hyde, K. W. Thilini Chethana, Jianhua Liu, Xinghong Li, Jiye Yan

**Affiliations:** 1 Beijing Municipal Key Laboratory for Environmental Friendly Management on Pests of North China Fruits, Institute of Plant and Environment Protection, Beijing Academy of Agriculture and Forestry Sciences, Beijing, 100097 People’s Republic of China; 2 College of Plant Protection, China Agricultural University, Beijing, 100193 People’s Republic of China; 3 Center of Excellence in Fungal Research, Mae Fah Luang University, Chiang Rai, 57100 Thailand; 4 Department of Biology, McMaster University, Hamilton, Ontario L8S 4K1 Canada; 5 School of Science, Mae Fah Luang University, Chiang, Rai 57100 Thailand

## Abstract

Downy mildew caused by *Plasmopara viticola* is one of the most devastating diseases of grapevines worldwide. So far, the genetic diversity and origin of the Chinese *P*. *viticola* population are unclear. In the present study, 103 *P*. *viticola* isolates were sequenced at four gene regions: internal transcribed spacer one (ITS), large subunit of ribosomal RNA (LSU), actin gene (ACT) and beta-tubulin (TUB). The sequences were analyzed to obtain polymorphism and diversity information of the Chinese population as well as to infer the relationships between Chinese and American isolates. High genetic diversity was observed for the Chinese population, with evidence of sub-structuring based on climate. Phylogenetic analysis and haplotype networks showed evidence of close relationships between some American and Chinese isolates, consistent with recent introduction from America to China via planting materials. However, there is also evidence for endemic Chinese *P*. *viticola* isolates. Our results suggest that the current Chinese *Plasmopara viticola* population is an admixture of endemic and introduced isolates.

## Introduction

Grape downy mildew caused by *Plasmopara viticola* (Berk & Curt.) Berl. &. De Toni., is one of the most troublesome diseases of grapevines worldwide^1^. This disease was first identified in North America^2^. It is hypothesised that the disease was accidentally introduced in to European grape vineyards in 1870s^3,4^ via grapevine cuttings, which were used to replace French vineyards destroyed by *Phylloxera* ^5^. Today, grape downy mildew is found in all grape-growing regions, causing major economic losses^1,2,6^. In China, grapevine downy mildew was first reported in 1899^7^. At present, this disease can be observed in almost all Chinese grape vineyards, causing significant yield losses. Many studies have been conducted to understand disease epidemiology and resistance development against *P*. *lasmopara viticola* in China. However, it is less known about the population structure of this pathogen.

Using the multiple gene genealogical approaches it has shown that the morphological simplicity and similarity among closely related fungal species often contained cryptic lineages^8^. Previous studies have shown that the causal agent of grape downy mildew, *P*. *viticola*, exhibits four cryptic lineages, which are structured according to the host species; *Vitis aestivlis*, *V*. *labrusca*, *V*. *riparia* and *V*. *vinifera* ^4^. To date, studies of cryptic species have mainly focused on their complex phylogeography, gene flow and hybridization processes^9^. For plant pathogens, it is also important to understand fitness differences within or between cryptic species to determine whether a strain may be more or less virulent than other strains, including parental strains (for hybrids)^10,11^. For some instance, hybridization has resulted in “superspecies,” which may exhibit an expanded host range^12^, surpassing parental strains and attaining the ability to exploit a new host^13^. Therefore, it is necessary to understand how pathogen populations vary with geography and host.

Several studies have been conducted to elucidate the genetic diversity and population structure of grape downy mildew pathogens using various molecular markers^4,7,14–17^. Yin *et al*.^7^, observed high genetic diversity in Chinese *P*. *viticola* populations using SSR markers. However, the origin of the Chinese *P*. *viticola* population and its relationship with the American population are currently unknown. The Chinese *P*. *viticola* population might have originated from an exotic introduction via introduced plant materials from its native country. Fungal pathogens have the ability to spread with planting materials worldwide^18^. To develop control measures, it is necessary to identify the routes of introduction and study the evolutionary potential of pathogen populations^19^.

Hence, the objectives of the present study were to understand the genetic diversity of *P*. *viticola* isolates in China and their evolutionary relationships with American *P*. *viticola* isolates. We used multilocus sequence data to determine the genetic diversity of *P*. *viticola* isolates obtained in nine grape-growing regions in China from two *Vitis* cultivars; *V*. *vinifera* and *V*. *vinifera* × *V*. *labrusca*. We sequenced and analysed the internal transcribed spacer (ITS), the large subunit of ribosomal RNA (LSU), a fragment of the gene encoding beta-tubulin (TUB) and a fragment of the actin gene (ACT) in 103 *P*. *viticola* strains. We hypothesized that if *P*. *viticola* was introduced to China via planting materials, there might be a close phylogenetic relationship between Chinese isolates and American isolates.

## Results

### *Plasmopara viticola* sampling and pathogen isolation

**Figure 1:**
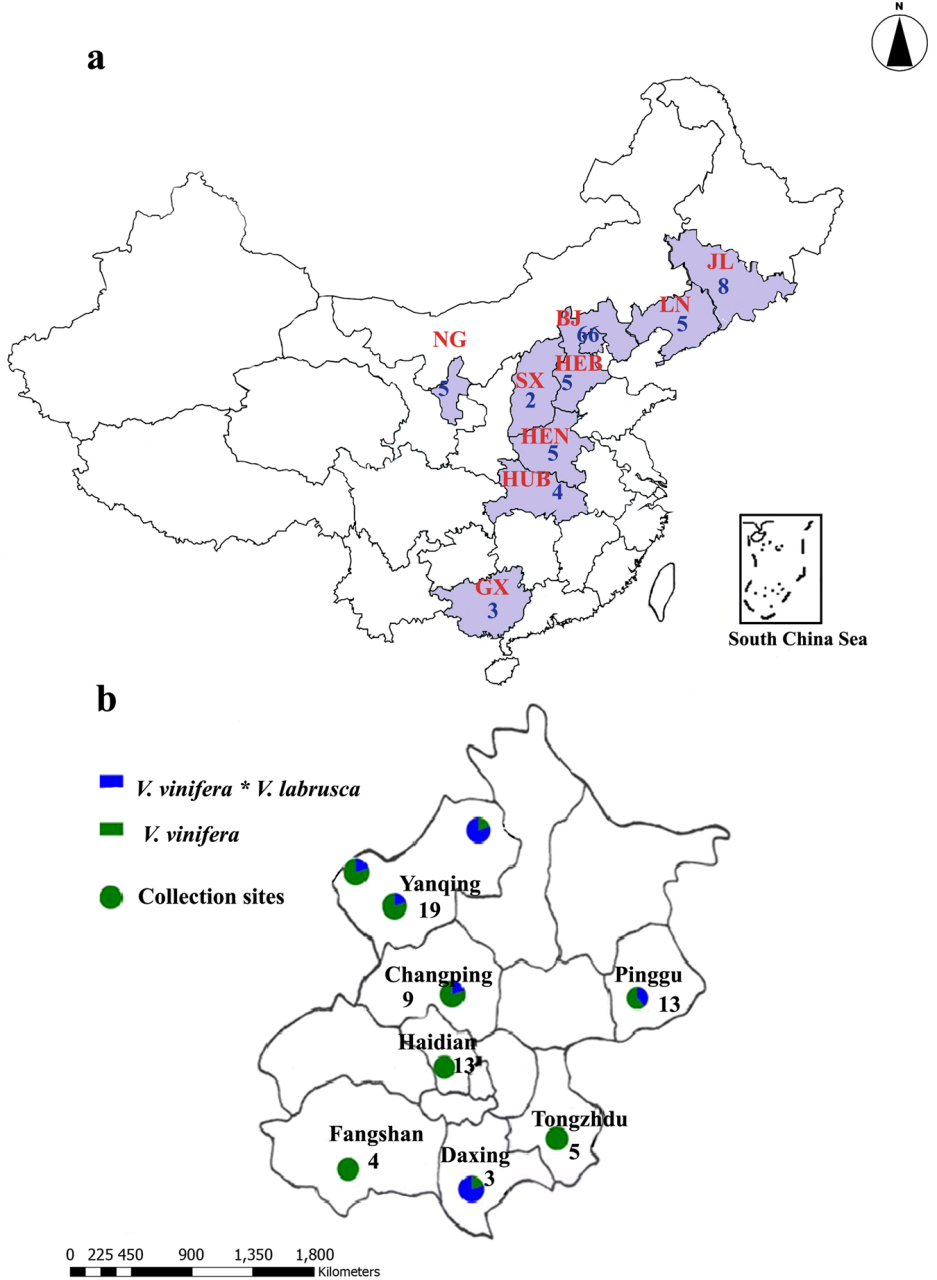
Geographical maps of *Plasmopara viticola* sample collecting sites created using SmartDraw 2016 (https://www.smartdraw.com/) and modified with Adobe photoshop CS6 (Adobe creative cloud, USA, www.adobe.com). (**a**) Map of China represents the nine sample collected provinces. (**b**) Beijing provincial map represents the sample-collected districts with respective number of isolates obtained. (Index BJ-Beijing, GX- Guangxi, HEB- Hebei, HUB- Hubei, HUN-Hunan, LN- Liaoning, JL- Jilin, NX- Ningxia and SH- Shanxi).

**Figure 2:**
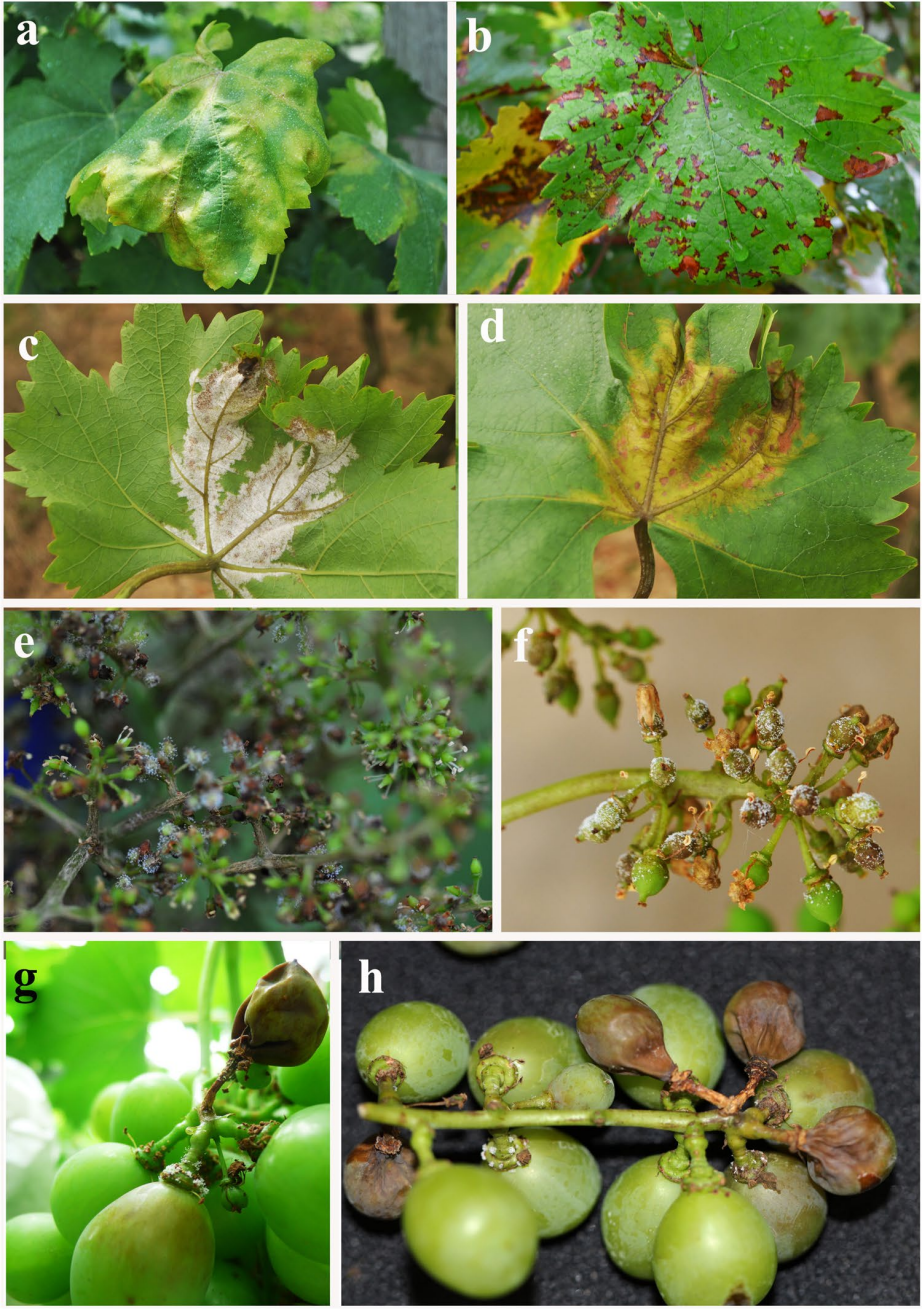
Field symptoms observed for grapevine downy mildew caused by *Plasmopara viticola*. (**a**) Oil spot symptom. (**b**) Old necrotic downy mildew leaf lesions. (**c**) Characteristic sporulation of pathogen from the underside of the leaf. (**d**) Necrotic lesions on the upper side of the leaf. (**e**,**f**) Characteristic sporulation on rachis. (**g**,**h**) Infections on young berries.

Sample collection sites and the respective number of isolates obtained from each of the nine provinces in China are given in Fig. 1a. Details on sample collection from different districts in Beijing and the number of isolates obtained from each district are shown in Fig. 1b. During the field survey, we observed grapevine leaves with oil spot symptoms, necrotic lesions and characteristic sporulation of the pathogen from the underside of the leaf (Fig. 2). Symptomatic leaves were used for pathogen isolation. Other than foliar symptoms, the most common symptoms observed in the field were characteristic sporulation on the rachis and infections on young berries (Fig. 2). In total, 103 *P*. *viticola* isolates were isolated via single-sporangium isolation (Supplementary Table S1). To understand the geographic variation within the Chinese *P*. *viticola* population, 103 isolates were divided into two climatological populations. The population “A” included the isolates obtained from subtropical climate region (Guangxi, Hubei and Hunan provinces). The population “B” included isolates obtained from temperate monsoon climate region (Beijing, Hebei, Jilin, Liaoning, Ningxia and Shanxi). PCR amplification for ACT, ITS, TUB and LSU gene regions using total genomic DNA as the template was successful for all isolates. The GenBank accession numbers for the American sequences used in this study are as follows: ACT: hap1–32 (JF897783–JF897815), ITS: hap1–4 (JF897779–JF897782), LSU: hap1–8 (JF897848–JF897855) and TUB: hap1–32 (JF897816–JF897847).

### Polymorphism and Population structure analysis

To understand the genetic diversity of Chinese *P*. *viticola*, analyses were conducted using DnaSp (V5)^20^. A summary of the genetic diversity and polymorphism indices for each gene region is given in Table 1. We observed highest number of alleles for the TUB (nine alleles), followed by ACT (four alleles), ITS (two alleles) and LSU (one allele). The TUB gave the highest average number of differences per site (0.005), whereas LSU gave the lowest (0.0). Haplotype diversity was highest for the TUB (0.71) and lowest for LSU (0.00). In addition to that, ITS and TUB gave positive Tajima’s D^21^ values as 0.48 and 1.0 respectively. To understand the gene flow and genetic differentiation, the Chinese *P*.*viticola* population was divided in to two climatological populations, subtropical population (A) and temperate monsoon population (B). Table 1 summarized the polymorphic data on the two sub-populations. The highest haplotype diversity (*h* = 0.68) and nucleotide diversity (pi = 2.80) was observed for the temperate population from TUB. The highest Fst between two populations was given by TUB as 0.28. To examine the evidence for recombination between the American and Chinese isolates, alleles from ACT and TUB were analyzed (ITS and LSU were excluded because of lack of polymorphism). For these regions, Hudson^22^ recombination parameter (R), overall genetic association by Kelly^23^ (Zns) and level of linkage disequilibrium (Za and ZZ)^23^ were calculated using equations available in DnaSP. In the ACT region, estimated R for gene was 0.001 and R between adjacent sites were 0.00. In this analysis, 990 pairwise comparisons were observed with 151 gametic types. Minimum numbers of recombination events^24^ were observed as 14 (Rm). We observed ZnS for ACT as 0.25. For ACT, Rozas *et al*.^25^, Za and ZZ values on recombination were 0.24 and -0.0097 respectively. In the TUB region, estimated R was 0.001 and R between adjacent sites were 0.00. In this analysis, 2,701 pairwise comparisons were observed with 464 gametic types. Minimum numbers of recombination events were observed as 25 (Rm). The overall genetic association (ZnS) was 0.2056 for the TUB region. Calculated Za and ZZ values were 0.2413 and 0.0357.

**Table 1:** Polymorphism and diversity data of *Plasmopara viticola* isolates obtain from china.

		n^a^	bp^b^	S^c^	nA^d^	hd^e^	pi^f^	D^g^
All	ITS	103	537	1	2	0.361	0.00081	1.00238
	LSU	102	693	0	1	0.00000	—	—
	ACT	101	435	3	4	0.1147	0.00133	−1.31401
	TUB	102	505	12	9	0.7109	0.00457	0.47827
A	ITS	12	523	1	2	0.30303	0.00068	—
	LSU	12	693	0	1	0.00000	—	—
	ACT	12	435	0	1	0.00000	0.00000	—
	TUB	12	505	2	2	0.48485	0.96970	—
B	ITS	79	523	1	2	0.38299	0.00086	—
	LSU	85	693	0	1	0.00000	—	—
	ACT	79	435	3	4	0.14508	0.19539	—
	TUB	85	505	12	8	0.68179	2.79776	—

^a^Sample size (n).

^b^Total number of sites (bp).

^c^Number of segregating sites (S).

^d^Number of alleles (nA).

^e^Haplotypic (allelic) diversity (hd).

^f^Average nucleotide diversity (pi).

^g^Tajima’s D (D).

### Phylogenetic analyses

**Figure 3:**
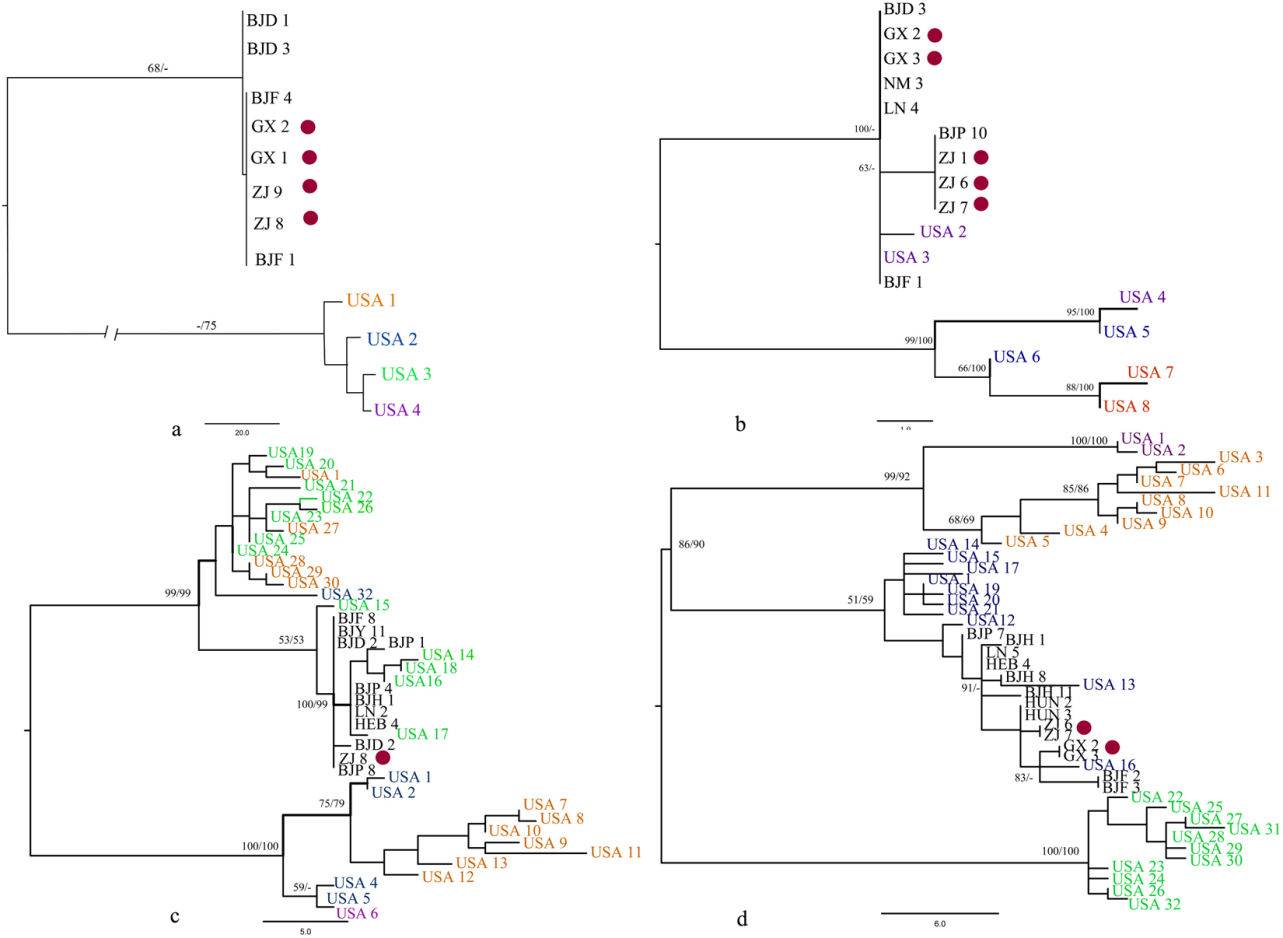
Maximum parsimony (MP) (using PAUP v4.0) trees obtained to reconstruct allele relationship for *Plasmopara viticola* isolates obtained from China and USA. (**a**) ITS tree. (**b**) LSU (28 S) tree. (**c**) actin (ACT) tree. (**d**) tubulin (TUB) tree. Bootstrap values given from more than 50% for MP and ML analysis. Bayesian posterior probabilities greater than 0.95 are given in bold. (Index GX: LN: Liaoning, Guangxi, ZJ: Jilin. From Beijing, **BJD**: Daxing, **BJF**: Fangshan, **BJH**: Haidian, **BJP**: Pinggu). For American isolates, *P*. *viticola* f. sp. *quinquefolia* isolates are given in purple colour, *P*. *viticola* f. sp. *aestivalis* isolates are given in blue colour *P*. *viticola* f. sp. *riparia* isolates are given in green colour *P*. *viticola* f. sp. *vinifera* isolates are given in orange colour. Dark pink circles represent the isolates obtained from Chinese traditional grapevine cultivars.

To understand the phylogenetic relationships between Chinese *P*. *viticola* and the American isolates, four phylogenetic trees were constructed based on each of the four gene regions (ITS, LSU, ACT and TUB). In this analysis, we excluded identical sequences from the Chinese isolates and used representative sequences from each haplotype given by polymorphism analysis. According to phylogenetic analysis, there are no distinct cladding patterns in Chinese *P*. *viticola* population based on geographical origin (Fig. 3). In polymorphic data analysis, ITS and LSU gave one haplotype for Chinese isolates; therefore to construct the phylogenetic tree, we used 10 random isolates from the Chinese isolates.

The phylogenetic tree constructed for ITS region is shown in Fig. 3a. Alignment for the ITS contained 537 bp with 413 constant characters, 2 parsimony uninformative characters and 122 parsimony informative characters. Parameters for the ITS phylogenetic tree are as follows: tree length (TL) = 133, consistency index (CI) = 0.985, retention index (RI) = 0.994, relative consistency index (RC) = 0.979, and homoplasy index (HI) = 0.015). In the phylogenetic analysis, we have observed two main clusters with 100 bootstrap values from MP and ML. The Chinese isolates were claded into a single cluster. Within this main cluster, isolates from Guangxi (GX 2 and 3) and Jilin (ZJ 8 and 9) were claded separately. The second cluster represented the isolates obtained from the America, in which four American haplotypes represented by independent nodes. The LSU phylogenetic analysis included 10 representative strains from China and eight sequences from America. The aligned sequences for the LSU gene region contained 686 bp with 660 characters being constant, 14 parsimony uninformative characters and 12 parsimony informative characters. The phylogenetic tree (TL = 27, CI = 1.00, RI = 1.00 RC = 1.00, HI = 0.00) constructed for the LSU gene is given in Fig. 3b. The LSU phylogenetic tree contained five independent clades for American haplotypes. The Chinese isolates cluster together with two American haplotypes, belonging to *P*. *viticola* fo.s. *quinquefolia*. Within the Chinese main clade, isolates obtained from Jilin province (ZJ 1, 6 and 7) clustered in a different sub-clade with 68 bootstrap values from MP analysis.

Phylogenetic tree for ACT gene region includes 10 sequences from China belonging to four haplotypes (Supplementary Table S1) and 32 sequences from America (Fig. 3c). The sequence alignment included 435 bp with 384 characters being constant, 11 parsimony uninformative characters and 40 parsimony informative characters. In the unrooted phylogenetic tree (TL = 93, CI = 0.645, RI = 0.918, RC = 0.592, HI = 0.355), 27 sequences from America were clustered into three clades (Purple, light blue and red clades in Fig. 3c). The remaining five sequences (belongs to *P*. *viticola* fo.s. *riparia* lineage) belonged to a different cluster with five Chinese isolates belongs to Chinese haplotype one. The TUB phylogenetic analysis (Fig. 3d) was also carried out on 14 sequences from China. These 14 sequences belong to nine haplotypes identified from polymorphic analysis. In this tree, 32 sequences from America were included. The aligned sequences contained 505 bp with 427 characters being constant, 6 parsimony uninformative characters and 72 parsimony informative characters. The TUB tree (TL = 136, CI = 0.610, RI = 0.925, RC = 0.565, HI = 0.390, Fig. 3d) showed that the 29 American sequences belonged to three main clusters. Three sequences from America belonging to the *P*.*viticola* f. sp. *aestivalis* lineage clustered with isolates from Chinese haplotype one (Supplementary Table S1). The TUB Sequence obtain from Jilin (ZJ 6) showed a similar clade pattern as ACT and LSU sequences by developing independent sub-clade.

### Haplotype relationship analysis

**Figure 4:**
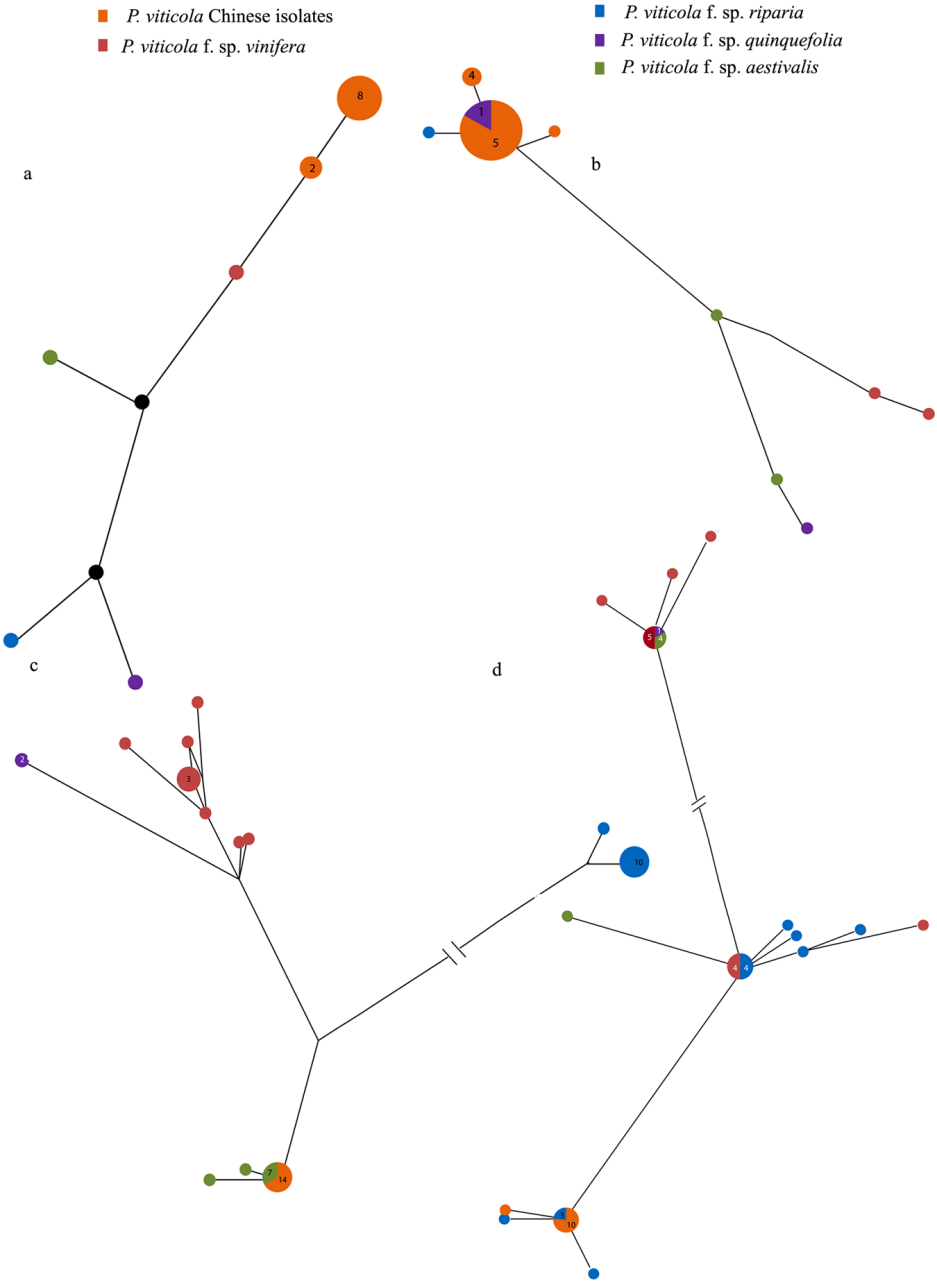
Median joining networks constructed for 136 *P*. *viticola* isolates obtained from China and America using Splitstree (v4). (**a**) ITS network. (**b**) LSU network (**c**). ACT network. (**d**) TUB network. Size of the bubble proportional to the number of isolates belongs to each node.

For each gene, median-joining haplotype networks were built under the K2P distance model (Fig. 4). The haplotypes obtained from polymorphic data analysis for Chinese isolates were analysed with the American isolates. Haplotype networks constructed for different gene regions of the Chinese haplotypes showed ancestry relationships with different cryptic lineages identified from the American population. Chinese haplotypes showed closer relationship to the *Plasmopara viticola* f. sp. *vinifera* in ITS analysis, *P*. *viticola* f. sp. *quinquefolia* in LSU analysis, *P*. *viticola* f. sp. *aestivalis* in ACT analysis and *P*. *viticola* f. sp. *riparia* in TUB and LSU analyses.

## Discussion

*Plasmopara viticola* is a heterothallic oomycetous that causes grapevine downy mildew^26^. This species constitutes one of the most important pathogens of grapevines (*Vitis vinifera* L.) worldwide. The disease symptoms include oil spots, leaves with the multiple lesions, and abscission, and severe infection resulted in defoliation^16^. Sporangia appear under the leaves and on other infected parts of the plants^2^. Bunches of young fruit turn brown and die rapidly when infected^16^. Older berries can be infected if the pathogen invades through the pedicel, causing symptoms referred to as brown rot or leather berry^2^. The disease epidemiology of this pathogen involves primary infection by oospores of the sexual stage of the pathogen^2,16^. This primary infection is followed by several asexual cycles of sporangium production, which results in colony infection and successful disease progression^2,16^.

China is one of the main grapevine-growing countries in the world. Chinese grape production has dramatically increased in the past 20 years^27^. According to world statistical data from 2014, the total area under grape vineyard cultivation is 7,573 Kha, for both table and wine grapes. China has become the second largest grape grower worldwide based on vineyard area (almost 800 Kha)^27^. However, in certain areas, grape production has decreased because of the continental climate, which allows disease epidemics of powdery and downy mildew to occur^28^. Therefore, it is important to understand the diversity and virulence factors of these pathogens for resistance breeding and the selection of fungicide-resistant strains. According to the literature, China is one of the centres reported for the origin of *Vitis* species^23,29,30^. It is predicted that 70 of the known species in this genus originated in China. Among them, the Chinese traditional cultivars are highly resistant to *Elsinoe ampelina* Shear^29^, *Coniella diplodiella* (Speg.) Petr. & Syd. 1927 and *Colletotrichum* Corda species^29,30^. In addition, characteristics such as flavour and breeding ability have garnered more attention for these traditional cultivars^29,31,32^.

Grapevine downy mildew was first reported in North America. However, the pathway through which *P*. *viticola* reached China has not been studied. In addition to the origin of the pathogen, it is important to understand the genetic diversity of *P*. *viticola* in Chinese vineyards to achieve sustainable management. To understand the genetic diversity and the origin of this pathogen, we collected *P*. *viticola* samples from nine grape-growing regions in China. However, majority of our samples were from Beijing since it was difficult to collect samples from long distances. Majority of single sporangia isolated from the traditional cultivars were failed. This might be due to the host specificity of the *P*. *viticola* isolated from Chinese traditional cultivars. Due to the host specificity and obligate biotrophic nature of the pathogen, we were unable to obtain samples outside of China. Thus, this study was only depended on the sequence data available in the NCBI GenBank and the sequences generated in the current study.

In population genetic studies, DNA sequencing approach facilitates data for both high level phylogenetic analyses among species and for the analyses of genetic variations among isolates within populations and species. We performed sequencing of the ITS, LSU, ACT and TUB gene regions for the Chinese populations. We also obtained sequences from North American isolates from NCBI GenBank. There were no considerable amounts of sequence data available for the European isolates in the NCBI. Therefore, we excluded the European isolates from the current study. We selected both conserved and non-functional DNA fragments for this analysis. Non-functional DNA regions are more informative than the conserved regions^33^. Therefore, we included TUB and ACT gene regions into the analysis. Previous studies have also confirmed that these gene regions are useful and informative to understand the population structure and genetic diversity of the *P*. *viticola* ^4^.

Genetic diversity of Chinese downy mildew population was calculated via haplotype diversity analysis. Haplotype diversity is a combined result of mutation, recombination, marker ascertainment and demography^34^. In the population investigated in the present study, we observed low haplotype and nucleotide diversity for ITS and LSU gene regions. However, ACT and TUB gave high haplotype diversity for Chinese *P*. *viticola* population. The sub population belongs to temperate monsoon region (A) gave higher haplotype diversity. The estimated nucleotide diversity also reflected similar results as above. Thus, it indicted high genetic diversity for Chinese *P*. *viticola* population. The average nucleotide diversity and estimated Theta (θ) per site were also high for TUB gene region. Yin *et al*.^7^ have studied *Plasmopara viticola* population using SSR markers to understand the population structure of grape downy mildew in China^7^. In that study, they observed a high level of genetic diversity in the *P*. *viticola* population. The current study confirms the high genetic diversity of Chinese grape downy mildew population using multilocus sequence data. In addition to Chinese studies, several studies have been conducted to understand the genetic diversity and population structure of the *Plasmopara viticola* ^4,35^. A study conducted by Rumbou and Gessler^35^ using microsatellite markers revealed that the *P*. *viticola* population from the Mediterranean islands exhibits limited variation and that disease epidemics in this region have been driven by multiple clonal expansions. The authors suggested that these findings might have been due to the isolation of Mediterranean islands from the main continent. A study conducted by Roxual *et al*.^4^, based on multiple genealogies and sporangia morphology showed that American *P*. *viticola* consists of a complex of four cryptic species, each of which is associated with different host plants. However, in the current study, we did not observe any host specificity but our major finding provide evidence for the climatological subpopulations of *Plasmopara viticola*.

Over the last few years, northern China has experienced increased temperatures^36^. Such increments may enhance the potential of pathogens to invade plants^10,36^. Since we observed high genetic diversity from the isolates obtained from northern China (temperate climatic region), the pathogen may have shown the potential to evolve with the changing environmental factors. Therefore it is necessary to alter the current management strategies with time^10,11^. To understand the gene flow and genetic differentiation between the two Chinese *P*. *viticola* subpopulations, we calculated nearest neighbour statistics (Snn). The Snn measures how often the most similar sequence or another sequence (nearest neighbour) is from the same distinct population^37^. This measurement is particularly powerful for small population sizes. If two populations exhibit a value near to one, this indicates that these two populations are highly differentiated, as almost every sequence would be similar to those from the same population^37–39^. A value of 0.5 is expected if populations are not genetically structured. In this case, the closest sequences would have an equal probability of being most similar to those from either population^37–39^. From our sequence data, we obtained Snn value higher than 0.5 for ACT and TUB (0.856; p = 0.00 and 0.769; p = 0.8). This suggested that the Chinese *P*. *viticola* population is genetically structured according to climatological origin. Other than that, Fst also can be used to measure genetic differentiation of a particular population.

Fst calculates the degree of genetic differentiation within a population based on allele frequencies^38^. It demonstrated a high potential for inbreeding within the groups examined in the present study^38^. The highest Fst was given by the TUB gene region as 0.277. Similar results were found in Yin *et al*.^7^, in which Chinese *P*. *viticola* population separated into three subpopulations according to geographical origin. Together, both studies suggest the existence of climatological subpopulations in *P*. *viticola* in China. However, the main objective of the present study was to understand the possible introduction pathways of *P*. *viticola* to the Chinese grape vineyards.

Tajima’s D indicates how much population variation can be sustained over time^3,21^. The positive D value reflects the selection maintaining variation, which might be resulted due to recent population contraction^3^. In the present study, we observed positive value for Tajima’s D for ITS and TUB (1.00 and 0.478). Within a population, controlled selection could result in population contraction^21^. This may be caused by the application of fungicides over the years. Many types of chemicals have been used to control downy mildew^5^. This might have led to develop resistant strains in the population, and many genetic types were lost from the main population^40^. Therefore, in the current population, we might only observe the individuals resulted from the resistant selection. In addition to the diversity analysis, use of multilocus sequence data gave us further information to track the origin of the Chinese *P*. *viticola* in China^33^.

To ensure the hypothesis of the possible introduction of *Plasmopara viticola* from North America, we analyzed Hudson and Kaplan^24^ index for the recombination. In here, we calculated the number of recombination events in the history of a sample of sequences (R) and the number of recombination events that can be parsimoniously inferred from a sample of sequences (Rm). Once the rate of recombination equals to the zero, R gives zero. We obtain R equals to zero for both ACT and TUB regions, which reflect zero recombination events, occurs in history for the current samples. Since the R is given the value on history of the sample, Rm, denote the minimum number of recombination events implied by the data using the four-gamete test. We observed 14 and 28 recombination events within 133 *P*. *viticola* isolates (From China and America). The positive ZZ value, which reflects intragenic recombination, has played an importance role in nucleotide variation^23^. We observed positive value on TUB region, as well as higher number of recombination events. Therefore, a recent recombination event might have happened between Chinese and American isolates, when they introduced to China with planting materials. To ensure the possible recombination between native isolates and Chinese isolates we performed phylogenetic analysis and haplotype network construction.

Haplotype networks allow to understand the coexistence of ancestral and derived haplotypes and it is an account for recombination. Therefore, haplotypes networks are preferable for intraspecific analyses^39–41^. In here, we used the most parsimonious network development in which the number of mutations separating each haplotype, with a parsimony probability of 95%^41,42^. In each network, ancestral haplotype was predicted based on rooting probability^39,43^. In the present study, we developed four haplotype network for ITS, LSU, ACT and TUB gene regions. In each network, we observed that there was an ancestral relationship between Chinese and American isolates. In phylogenetic analysis, similar cladding patterns were observed. This result supports our hypothesis on the introduction of grape downy mildew pathogens to China from Northern America. In addition to that, we observed several isolates from China, which showed independent clustering in phylogenetic analysis. Those isolates were obtained from Chinese traditional grapevine cultivars. These results suggested that current *P*. *viticola* population in China might have an admixture population, where the population is made up with both endemic and introduced isolates. The endemic evolution is possible with the cross breeding over many generations. Chinese traditional grape cultivars are frequently used in breeding programs to develop high quality and pathogen resistant cultivars^27^. To overcome this selection pressure, pathogen might have evolved from generation to generation. In addition to that, the planting materials exchange between countries might be lead to introduce some foreign strains to the China.

The present study was conducted to understand the genetic diversity and evolutionary pathways of *Plasmopara viticola* population in China. We used DNA sequencing at multiple loci as the marker of choice in the present study. Using multiple genealogical approaches, we observed high genetic diversity in Chinese *P*. *viticola* population. Our results demonstrate that the *P*. *viticola* population in China sub divided according to the climatological regions. Since most samples were collected from *Vitis vinifera*, we cannot provide conclusion on host specificity of Chinese *P*. *viticola* population. Therefore, to understand the host specificity related pathogenicity, further studies are required. The *Plasmopara viticola* population in China was observed to have selection maintaining variation by giving positive Tajima’s D values. It is predicted that, this is due to resistance development over recent years with the excess use of chemical fungicides or the resistance breeding. Observing possible recombination between American and Chinese isolates supported our hypothesis on possible introduction from Native American population. Both phylogenetic and haplotype networks showed possible ancestral relationship with each other supporting the hypothesis of possible pathogen introduction form native population. However, *Plasmopara viticola* isolates obtained from traditional Chinese grape cultivars were observed to be genetically distant in above the analyses. Therefore, we concluded that the current *Plasmopara viticola* population in China is a combination of endemic and introduced strains.

## Materials and Methods

### *Plasmopara* viticola sample collection

The sample collection sites were located in nine provinces in China: Beijing (BJ), Guangxi (GX), Hebei (HEB), Hubei (HUB), Hunan (HUN), Liaoning (LN), Jilin (JL), Ningxia (NX) and Shanxi (SX). In Beijing, samples were collected from seven districts: Changping (BJC), Daxing (BJD), Fangshan (BJF), Haidian (BJH), Pinggu (BJP), Tongzhou (BJT) and Yanquing (BJY). Symptomatic tissues were collected from both *V*. *vinifera* and *V*. *vinifera* × *V*. *labrusca* hybrids. Diseased samples were collected into plastic bags with sterilized tissues, which were dipped in distilled water to maintain humid conditions.

### Fungal isolation and DNA extraction

Infected leaves were incubated for 2–4 days at 18 °C under a photoperiod of 16 h day/8 h night. To isolate the pathogen, the “leaf disc incubation” method was used. A humid container was constructed using two 90 diameter sterilized Petri dishes, in which the petri dishes were stacked upon one another. Filter paper soaked in 8 ml of sterile water was added to the bottom dish. To inoculate the pathogen, leaves from susceptible cultivars (Red Globe and Pinger) were used. Summer Black leaves used as the control. Selected young leaves were examined under a dissecting microscope to ensure that no downy mildew had fallen on the foliage or mycelia. Selected leaves were surface sterilized with a 1% (mass percent concentration) sodium hypochlorite solution for 30 sec, followed by three washes with sterilized distilled water. Excess water on the leaf blades was absorbed with sterile filter paper. Once disinfection was completed, 2 cm diameter cork borer was used to obtain leaf discs from the middle of the leaf, avoiding the main vein and the larger lateral veins. Finally, the flat leaf discs were placed in the prepared humidifiers, with five leaf discs per dish. Under a dissecting microscope, an acupuncture needle (size Ф 0.25 × 40 mm) was used to transfer newly generated sporangiophores from the incubated leaves onto leaf discs. For each grape cultivar, 50 leaf discs were inoculated. To obtain pure cultures, single-sporangium isolation was repeated several times. After leaf disc cultures obtained from a single spore were established, they were used for colony formation. For DNA extraction, approximately 100 mg of the mycelium was collected. The DNeasy Plant Mini Kit (Qiagen, Germany) was used to extract total genomic DNA.

### PCR amplification

PCR amplification of total genomic DNA was conducted for four gene regions: internal transcribed spacer one (ITS), the large subunit of ribosomal RNA (LSU), a fragment of the gene encoding beta-tubulin (TUB) and a fragment of the actin gene (ACT). The primer sequences and annealing temperatures employed for PCR are given in Table 2. PCR was carried out in a final volume of 25 μl. The PCR mixture consisted of 1 μl of genomic DNA, 2 mM MgCl_2_, 5 μM of each deoxynucleoside triphosphate (dNTP), 1 μl of each primer and 0.3 U of Taq Silver star DNA polymerase in 1x reaction buffer. The thermo cycling conditions were as follows: 95 °C for 4 min, followed by 40 cycles of 95 °C for 40 s, 52–58 °C for 45 s, and 72 °C for 90 s, with a final step at 72 °C for 10 min. Following the PCR amplification, products were visualised on 1% agarose gel under UV light using a Gel Doc^TM^ XR + Molecular Imager (Bio-Rad, USA). Sequencing of the positive amplicons was carried on a Sun-biotech Company Sequencer (Beijing, China).

**Table 2:** Specific primers used for phylogenetic analysis of *Plasmopara viticola* in the study.

Gene	Primer Name	Sequences (5′-3′)	Product size
			(bp)
ITS1	ITS1	CTTGGCATTTCATCCTTCCGT	708
	ITS2	AGCCAACCATACCGCAAATC	
28 S	28S1	GCATATCAATAAGCGGAGGAAAAG	699
	28S4	GGTCCGTGTTTCAAGACGG	
actin a	ACT-F	GCTGACGAAGACGTTCAGG	435
	ACT-R	TGTAATCCGTCAGGTCACGA	
b-tubulin	Tub-F	CACTGTCGTTGAGCCCTACA	505
	Tub-R	AAACGTGGTGCTCATTTTCA	

### Sequence assembly

The chromatograms of the sequences were checked using BioEdit (v5)^44^ to ensure quality sequences. Consensus sequences were generated using DNAStar (v5.1). Sequences were analysed using the GenBank BLASTn search engine of the National Center for Biotechnology Information (NCBI). The sequence data have been deposited in GenBank (ITS: KF131651–KF131679; KY933859–KY933921; LSU: KF160831–KF160858, KY933922–KY933984; TUB: KF160791–KF160819, KY933985–KY934046; ACT: KF160751–KF160779, KY933798–KY933858). The sequences for American isolates were obtained from NCBI GenBank^4^.

### Polymorphism and Population structure analysis

The combined sequence dataset including the Chinese isolates was evaluated to understand genetic diversity. DnaSP (V5)^20^ was used to calculate the following parameters: number of haplotypes, haplotype diversity and pairwise nucleotide diversity (Л). To overcome population size effects parameters were obtained after 1,000 repetitions, and the median estimate for each parameter was recorded. Tajima’s D^21^ was calculated to test for departure from an equilibrium natural model of evolution. DnaSP (v5) was used to measure the Tajima’s D value, with a permutation test of 1,000 replicates. To understand the population structure of *P*. *viticola*, nearest statistics (Snn) and F statistics values were calculated. To calculate the recombination between alleles, Hudson^22^ calculation and to identify the linkage disequilibrium Kelly^23^ calculation were performed.

### Phylogenetic analysis

The individual sequence sets belongs to ITS, LSU, ACT and TUB were aligned using the default settings of MAFFT (v7) (http://mafft.cbrc.jp/alignment/server/)^45^. If necessary, manually aligned the files using BioEdit^44^. Phylogenetic relationships among individuals were inferred via maximum parsimony (MP) implemented in PAUP (v4.0)^46^, maximum likelihood (ML) in RAXM^47^ and Bayesian analyses in MrBayes (v3.0b4)^48^. MP genealogies were constructed using the heuristic search function with 1000 random addition replicates, performing tree bisection and reconstruction (TBR) using a branch-swapping algorithm^49^. Branches of zero length were collapsed, and all multiple parsimonious trees were saved. Descriptive statistics including tree length (TL), consistency index (CI), retention index (RI), relative consistency index (RC) and homoplasy index (HI) were calculated. Differences between the trees inferred under different optimality criteria were evaluated using Kishino-Hasegawa tests (KHT)^50^. Maximum likelihood analysis was conducted in raxmlGUI (v0.9b2)^47^ with 1000 non-parametric bootstrapping iterations, using the general time reversible model (GTR) with a discrete gamma distribution. Tree with the final likelihood value of −12360.77 was selected as the best scoring tree and the replicates were plotted relative to that. Suitable models for Bayesian analysis were obtained from JModel Test (v2.1.4)^51^. In the Bayesian analyses, Markov Chain Monte Carlo (MCMC) chains were run from random trees for 10,000,000 generations and sampled every 1,000 generation. The temperature value was lowered to 0.15, burn-in was set to 0.25, and the run was automatically stopped as soon as the average standard deviation of split frequencies was less than 0.01. Resulted phylogenetic trees were viewed using Treeview (v1.6.6)^52^.

### Haplotype relationship analysis

The aligned sequence dataset, which included isolates from China and America, was employed to develop haplotype networks. The haplotype networks were constructed using Splitree (v4.13)^53^. The median-joining network was built with the K2P distance model for each gene region. Statistically significant splits were obtained at a 95% confidence level with 1,000 bootstrap replicates. To identify possible recombination events, the PHI index was calculated.

## Electronic supplementary material

Supplementary table S1
