# Supplementary material for: Multiple gene genealogy reveals high genetic diversity and evidence for multiple origins of Chinese Plasmopara viticola population
Source: Sci Rep. 2017 Dec 11;7:17304. doi: 10.1038/s41598-017-17569-7 (PMC5725484; doi:10.1038/s41598-017-17569-7)
Supplement: Supplementary file 1 — Supplementary table S1 [file 41598_2017_17569_MOESM1_ESM.pdf]

Multiple gene genealogy reveals high genetic diversity and evidence for multiple origins of Chinese *Plasmopara viticola* population

Wei Zhang [1,3+], Ishara S. Manawasinghe[1,2+], Wensheng Zhao[3], Jianping Xu[4], Siraprapa Brooks[5], Xueyan Zhao[1], Kevin D. Hyde[2], K.W. Thilini Chethana[1,2], Jianhua Liu[1], Xinghong Li[1]*, Jiye Yan[1]*

Supplementary Table S 1

Details of the *Plasmopara viticola* isolates used in the study

Isolate Number	Grape cultivar	Location	GenBank No			
			ITS	ACT	TUB	LSU
JZB3100001[B, a,X,2]	*V. vinifera*	PingGu, Beijing, China	KY933859	KY933798	KY933985	KY933922
JZB3100002[B, a,Y,2]	*V. vinifera*	PingGu, Beijing, China	KY933860	KY933799	KY933986	KY933923
JZB3100003[B,b,W,2]	*V. vinifera*	PingGu, Beijing, China	KY933861	KY933800	KY933987	KY933924
JZB3100004[B,b,Z,2]	*V. vinifera*	PingGu, Beijing, China	KY933862	KY933801	KY933988	KY933925
JZB3100005[B,b,W,1]	*V. vinifera*	PingGu, Beijing, China	KY933863	KY933802	-	KY933926
JZB3100006[B,b,W,2]	*V. vinifera*	PingGu, Beijing, China	KY933873	KY933812	KY933998	KY933936
JZB3100007[B,b,W,5]	*V. vinifera*	PingGu, Beijing, China	KY933905	-	KY934030	KY933968
JZB3100008[B,b,W,3]	*V. vinifera*	PingGu, Beijing, China	KY933906	KY933843	KY934031	KY933969
JZB3100009[B,b,W,3]	*V. vinifera*	PingGu, Beijing, China	KY933907	KY933844	KY934032	KY933970
JZB3100010[B,b,W,7]	*V. vinifera*	PingGu, Beijing, China	KY933909	KY933846	KY934034	KY933972
JZB3100011[B,b,W,1]	*V. vinifera*×*V. labrusca*	PingGu, Beijing, China	KF131672	KF160772	KF160812	KF160851
JZB3100012[B, b,W,1]	*V. vinifera*	PingGu, Beijing, China	KF131673	KF160773	KF160813	KF160852
JZB3100013[B,b,W,1]	*V. vinifera*	PingGu, Beijing, China	KF131674	KF160774	KF160814	KF160853
JZB3100014[B,b,W,3]	*V. vinifera*	Changping, Beijing, China	KY933864	KY933803	KY933989	KY933927
JZB3100015[B,b,W,3]	*V. vinifera*	Changping, Beijing, China	KY933865	KY933804	KY933990	KY933928
JZB3100016[B,b,W,2]	*V. vinifera*	Changping, Beijing, China	KY933869	KY933808	KY933994	KY933932
JZB3100017[B,b,W,2]	*V. vinifera*	Changping, Beijing, China	KY933870	KY933809	KY933995	KY933933
JZB3100018[B,a,W,1]	*V. vinifera*×*V. labrusca*	Changping, Beijing, China	KF131649	KF160749	KF160789	KF160829
JZB3100019[B,b,W,1]	*V. vinifera*	Changping, Beijing, China	KF131650	KF160750	KF160790	KF160830
JZB3100020[B,b,W,1]	*V. vinifera*×*V. labrusca*	Changping, Beijing, China	KF131669	KF160769	KF160809	KF160848

JZB3100021 [B,b,W,1]	*V. vinifera*	Changping, Beijing, China	KF 131670	KF160770	KF160810	KF160849
JZB3100022 [B,b,W,1]	*V. vinifera*	Changping, Beijing, China	KF131671	KF160771	KF160811	KF160850
JZB3100023 [B,b,W,3]	*V. vinifera*	YanQing, Beijing, China	KY933867	KY933806	KY933992	KY933930
JZB3100024 [B, a,W,3]	*V. vinifera*	YanQing, Beijing, China	KY933868	KY933807	KY933993	KY933931
JZB3100025 [B,b,W,2]	*V. vinifera*	YanQing, Beijing, China	KY933871	KY933810	KY933996	KY933934
JZB3100026 [B,b,W,2]	*V. vinifera*	YanQing, Beijing, China	KY933872	KY933811	KY933997	KY933935
JZB3100027 [B,b,W,1]	*V. vinifera*	YanQing, Beijing, China	KF131643	KF160743	KF160783	KF160826
JZB3100028 [B,b,W,1]	*V. vinifera*	YanQing, Beijing, China	KF131644	KF160744	KF160784	KF160827
JZB3100029 [B,b,W,1]	*V. vinifera*×*V. labrusca*	YanQing, Beijing, China	KF131645	KF160745	KF160785	KF160822
JZB3100030 [B,b,W,1]	*V. vinifera*	YanQing, Beijing, China	KF131657	KF160757	KF160797	KF160837
JZB3100031 [B,a,W,1]	*V. vinifera*	YanQing, Beijing, China	KF131658	KF160758	KF160798	KF160838
JZB3100032 [B,b,W,1]	*V. vinifera*×*V. labrusca*	YanQing, Beijing, China	KF131659	KF160759	KF160799	KF160839
JZB3100033 [B,b,W,1]	*V. vinifera*	YanQing, Beijing, China	KF131660	KF160760	KF160800	KF160840
JZB3100034 [B,b,W,1]	*V. vinifera*	YanQing, Beijing, China	KF131661	KF160761	KF160801	KF160841
JZB3100035 [B,a,W,1]	*V. vinifera*	YanQing, Beijing, China	KF131662	KF160762	KF160802	KF160842
JZB3100036 [B,a,W,1]	*V. vinifera*	YanQing, Beijing, China	KF131663	KF160763	KF160803	KF160843
JZB3100037 [B,b,W,1]	*V. vinifera*	YanQing, Beijing, China	KF131664	KF160764	KF160804	KF160844
JZB3100038 [B, a,W,1]	*V. vinifera*	YanQing, Beijing, China	KF131665	KF160765	KF160805	-
JZB3100039 [B, a,W,1]	*V. vinifera*×*V. labrusca*	YanQing, Beijing, China	KF131666	KF160766	KF160806	KF160845
JZB3100040 [B,b,W,1]	*V. vinifera*	YanQing, Beijing, China	KF131667	KF160767	KF160807	KF160846
JZB3100041 [B, a,W,1]	*V. vinifera*	YanQing, Beijing, China	KF131668	KF160768	KF160808	KF160847
JZB3100042 [B,b,Z,4]	*V. vinifera*	Haidian, Beijing, China	KY933866	KY933805	KY933991	KY933929

JZB3100043 [B,b,W,1]	*V. vinifera*	Haidian, Beijing, China	KY933895	KY933833	KY934020	KY933958
JZB3100044 [B,b,W,2]	*V. vinifera*	Haidian, Beijing, China	KY933896	KY933834	KY934021	KY933959
JZB3100045 [B,b,W,2]	*V. vinifera*	Haidian, Beijing, China	KY933897	KY933835	KY934022	KY933960
JZB3100046 [B,b,W,2]	*V. vinifera*	Haidian, Beijing, China	KY933899	KY933836	KY934023	KY933961
JZB3100047 [B,a,W,2]	*V. vinifera*	Haidian, Beijing, China	KY933899	KY933837	KY934024	KY933962
JZB3100048 [B,a,W,2]	*V. vinifera*	Haidian, Beijing, China	KY933900	KY933838	KY934025	KY933963
JZB3100049 [B,b,W,6]	*V. vinifera*	Haidian, Beijing, China	KY933908	KY933845	KY934033	KY933971
JZB3100050 [B,a,W,1]	*V. vinifera*	Haidian, Beijing, China	KF131640	KF220645	KF160780	KF160823
JZB3100051 [B,,a,W,1]	*V. vinifera*	Haidian, Beijing, China	KF131641	KF160742	KF160781	KF160824
JZB3100052 [B,b,W,1]	*V. vinifera*	Haidian, Beijing, China	KF131642	KF220644	KF160782	KF160825
JZB3100053 [B,b,W,1]	*V. vinifera*	Haidian, Beijing, China	KF131655	KF160755	KF160795	KF160835
JZB3100054 [B,b,W,1]	*V. vinifera*	Haidian, Beijing, China	KF131656	KF160756	KF160796	KF160836
JZB3100055 [B,b,W,1]	*V. vinifera*	Fangshan, Beijing, China	KF131651	KF160751	KF160791	KF160831
JZB3100056 [B,b,W,1]	*V. vinifera*	Fangshan, Beijing, China	KF131652	KF160752	KF160792	KF160832
JZB3100057 [B,b,W,1]	*V. vinifera*	Fangshan, Beijing, China	KF131653	KF160753	KF160793	KF160833
JZB3100058 [B,b,W,1]	*V. vinifera*	Fangshan, Beijing, China	KF131654	KF160754	KF160794	KF160834
JZB3100059 [B,b,W,1]	*V. vinifera*	TongZhou, Beijing, China	KF131646	KF160746	KF160786	KF160820
JZB3100060 [B,b,W,1]	*V. vinifera*	TongZhou, Beijing, China	KF131647	KF160747	KF160787	KF160820
JZB3100061 [B,b,W,1]	*V. vinifera*	TongZhou, Beijing, China	KF131648	KF160748	KF160788	KF160828
JZB3100062 [B,b,W,1]	*V. vinifera*	TongZhou, Beijing, China	KF131675	KF160775	KF160815	KF160854
JZB3100063 [B,b,W,1]	*V. vinifera*	TongZhou, Beijing, China	KF131676	KF160776	KF160786	KF160855
JZB3100064 [B,b,W,1]	*V. vinifera*	DaXing, Beijing, China	KF131677	KF160777	KF160817	KF160856

JZB3100065 [B, a,W,1]	*V. vinifera×V. labrusca*	DaXing, Beijing, China	KF131678	KF160778	KF160818	KF160857
JZB3100066 [B, a,W,1]	*V. vinifera*	DaXing, Beijing, China	KF131679	KF160779	KF160819	KF160858
JZB3100067[A,b,W,2]	*V. vinifera*	Guanxi, China	KY933874	KY933813	KY933999	KY933937
JZB3100068 [A,b,W,2]	*V. vinifera*	Guanxi, China	KY933875	KY933814	KY934000	KY933938
JZB3100069 [A,b,W,2]	*V. vinifera*	Guanxi, China	KY933876	KY933815	KY934001	KY933939
JZB3100070 [B, b,W,2]	*V. vinifera*	Ningxia, China	KY933877	KY933816	KY934002	KY933940
JZB3100071 [B,b,W,2]	*V. vinifera*	Ningxia, China	KY933878	KY933817	KY934003	KY933941
JZB3100072 [B, a,W,2]	*V. vinifera*	Ningxia, China	KY933879	KY933818	KY934004	KY933942
JZB3100073 [B,b,W,2]	*V. vinifera*	Ningxia, China	KY933880	KY933819	KY934005	KY933943
JZB3100074 [B,b,W,2]	*V. vinifera*	Ningxia, China	KY933880	KY933820	KY934006	KY933944
JZB3100075 [B,b,W,2]	*V. vinifera*	Hebei, China	KY933882	KY933821	KY934007	KY933945
JZB3100076 [B, a,W,2]	*V. vinifera*	Hebei, China	KY933883	-	KY934008	KY933946
JZB3100077 [B, a,W,2]	*V. vinifera*	Hebei, China	KY933884	KY933822	KY934009	KY933947
JZB3100078 [B,b,W,3]	*V. vinifera*	Hebei, China	KY933904	KY933842	KY934029	KY933967
JZB3100079 [B,b,Z,3]	*V. vinifera*	Hebei, China	KY933901	KY933839	KY934026	KY933964
JZB3100080 [B,a,W,2]	*V. vinifera*	Shanxi, China	KY933885	KY933823	KY934010	KY933948
JZB3100081 [B,a,W,2]	*V. vinifera*	Shanxi, China	KY933886	KY933824	KY934011	KY933949
JZB3100082 [A,a,W,2]	*V. vinifera*	NM,	KY933887	KY933825	KY934012	KY933950
JZB3100083 [A, b,W,2]	*V. vinifera*	NM,	KY933888	KY933826	KY934013	KY933951
JZB3100084 [A, a,W,2]	*V. vinifera*	NM,	KY933889	KY933827	KY934014	KY933952
JZB3100085 [A,b,W,2]	*V. vinifera*	NM,	KY933890	KY933828	KY934015	KY933953
JZB3100086 [B,b,W,2]	*V. vinifera*	Liaoning, China	KY933892	KY933830	KY934017	KY933955

JZB3100087 [B,b,Z,2]	*V. vinifera*	Liaoning, China	KY933893	KY933831	KY934018	KY933956
JZB3100088 [B,b,W,2]	*V. vinifera*	Liaoning, China	KY933894	KY933832	KY934019	KY933957
JZB3100089 [B,b,W,3]	*V. vinifera*	Liaoning, China	KY933902	KY933840	KY934027	KY933965
JZB3100090 [B,b,W,3]	*V. vinifera*	Liaoning, China	KY933903	KY933841	KY934028	KY933966
JZB3100091 [A,b,W,8]	*V. vinifera*	Hunan, China	KY933910	KY933847	KY934035	KY933973
JZB3100092 [A,b,W,8]	*V. vinifera*	Hunan, China	KY933911	KY933848	KY934036	KY933974
JZB3100093 [A,b,W,8]	*V. vinifera*	Hunan, China	KY933912	KY933849	KY934037	KY933975
JZB3100094 [A,b,W,8]	*V. vinifera*	Hunan, China	KY933913	KY933850	KY934038	KY933976
JZB3100095 [A,b,W,1]	*V. vinifera*	Hunan, China	KY933891	KY933829	KY934016	KY933954
JZB3100096 [B,b,W,9]	*V. vinifera*	ZIjiang, China	KY933914	KY933851	KY934039	KY933977
JZB3100097 [B b,W,9]	*V. vinifera*	ZIjiang, China	KY933915	KY933852	KY934040	KY933978
JZB3100098 [B,b,W,9]	*V. vinifera*	ZIjiang, China	KY933916	KY933853	KY934041	KY933979
JZB3100099 [B,b,W,9]	*V. vinifera*	ZIjiang, China	KY933917	KY933854	KY934042	KY933980
JZB3100100 [B,b,W,9]	*V. vinifera*	ZIjiang, China	KY933918	KY933855	KY934043	KY933981
JZB3100101 [B,b,W,9]	*V. vinifera*	ZIjiang, China	KY933919	KY933856	KY934044	KY933982
JZB3100102 [B,b,W,9]	*V. vinifera*	ZIjiang, China	KY933920	KY933857	KY934045	KY933983
JZB3100103 [B,b,W,9]	*V. vinifera*	ZIjiang, China	KY933921	KY933858	KY934038	KY933984

A subtropical population and B temperate population, a-b haplotypes obtained from ITS, w-z haplotypes obtained from ACT and 1-9 haplotypes obtained from TUB. The bold letters indicates isolates used in Phylogenetic analysis and haplotype network construction.
